# Phenotypic features and molecular study of airborne *Penicillium* species isolated in the northern part of the Persian Gulf, Bushehr, Iran

**DOI:** 10.18502/cmm.7.2.7035

**Published:** 2021-06

**Authors:** Behrouz Naeimi, Iman Mohsenifard, Saham Ansari, Farzaneh Sadeghzadeh, Gholamreza Khamisipour, Sina Dobaradaran, Fatemeh Faraji Ghasemi, Bahram Ahmadi

**Affiliations:** 1 Department of Medical Laboratory Sciences, Faculty of Paramedical, Bushehr University of Medical Sciences, Bushehr, Iran; 2 The Bachelor of Medical Laboratory Sciences, Student Research Committee, Bushehr University of Medical Sciences, Bushehr, Iran; 3 Department of Parasitology and Mycology, School of Medicine, Shahid Beheshti University of Medical Sciences, Tehran, Iran; 4 Department of Hematology, Faculty of Allied Medicine, Bushehr University of Medical Sciences, Bushehr, Iran; 5 Systems Environmental Health and Energy Research Center, The Persian Gulf Biomedical Sciences Research Institute, Bushehr University of Medical Sciences, Bushehr, Iran; 6 Department of Environmental Health Engineering, Faculty of Health and Nutrition, Bushehr University of Medical Sciences, Bushehr, Iran

**Keywords:** Beta-tubulin, Bushehr, *Penicillium*

## Abstract

**Background and Purpose:**

The main environmental saprobes, such as *Penicillium*, play an essential role in natural ecosystems as economically, ecologically, and medically important microorganisms. Biodiversity of this genus has not been described in Bushehr city, Iran. The present study is based on air biodiversity of *Penicillium* species on culture-dependent approach and culture-independent technique using partial b-tubulin sequences.

**Materials and Methods:**

By using active sampling with a high volume air sampler, a total of 157 *Penicillium* isolates were selected and screened for phenotypic characters. For the purposes of the study, 46 strains representative of 11 morphological species were selected and identified by molecular analysis.

**Results:**

Based on the findings, *P. crustosum* (18 isolates, 39.1%) and *P. chrysogenum* (15 isolates, 32.6%) were the most common isolated species, followed by *P. brevicompactum*, *P. rubens*, *P. citrinum*, *P. italicum* (each 2 isolates, 4.3%), *P. olsonii*, *P. expansum*, *P. griseofulvum*, *P. palitans*, and *P. polonicum* (each 1 isolate, 2.1%). Except for *P. chrysogenum* and *P. expansum* with floccose colony texture, the rest of the isolated species had velutinous texture.

**Conclusion:**

This is the first report in southern Iran to identify a large number of *Penicillium* strains isolated from air samples, showing *P. crustosum* and *P. chrysogenum* as the most common isolated species.

## Introduction

The genus *Penicillium* is classified in the family Aspergillaceae (Eurotiales) which is divided into two subgenera, 28 sections, and 354 known species with a comprehensive distribution with a wide range of ecological habitats [ 1 - 3 ]. They are the main environmental saprobes playing a significant ecological role as a decomposer of organic materials in the soil and vegetation [ 3 , 4 ]. Some species 

are able to grow in excessive environments, such as lower or higher temperature, higher salt and sugar concentrations, water deficiency, low oxygen levels, and low pH [ 1 , 5 - 7 ]. 

*Penicillium spp*. are also well-known for their capability to generate a significant diversity of secondary metabolites. Secondary metabolites are small molecules that function as toxins, signaling molecules, enzymes, antibiotics, pigments, and extrolites that are either harmful or beneficial to human welfare [ 2 , 8 - 10 ]. Aerosol scattering of fungi may cause a variety of adverse health effects, especially lower airway inflammatory response [ 11 ]. 

The city of Bushehr is located in the southwest of Iran that due to its proximity to the Persian Gulf, is hot and humid. Due to repeated dust storms in recent years and its rich resources of oil, gas, and petrochemical industries, the city has air pollution that may lead to an increased risk of allergic diseases associated with air- and dust-borne fungal species [ 12 , 13 ]. Therefore, considering the lack of information on *Penicillium* diversity in Bushehr, the present study aimed to identify species of *Penicillium* isolates according to culture-based morphology and molecular technique using partial b-tubulin (BT2) sequences as the gold standard target.

## Materials and Methods

### Samples

Active sampling was performed using a large volume air sampler. The 24-h outdoor samples were collected on quartz fiber filters (8×10 in) using a high volume air sampler at a flow rate of 1.42-1.58 m/min. The high volume air sampler was installed on the highest point of rooftop of the building (with a height of 3-4.5 m above the ground) [ 14 ]. For fungal isolation, the collected filter samples were divided into equal parts (1 cm fractions) and placed on Sabouraud dextrose agar (SDA) (Merck, Germany) culture plates and incubated at 25 °C for 7 days. The colonies suspected as *Penicillum* were selected and sub-cultured. Identification at genus level was done by using both molecular and morphological approaches. 

### DNA extraction and molecular identification

For molecular identification of the isolates, fungal genomic DNA was extracted from a single isolated colony using glass bead disruption [ 15 ]. The BT2 gene amplification was performed using the primers Bt2a and Bt2b [ 16 ]. Amplified partial b-tubulin gene region products were purified and sequenced by a DNA sequencing service (Macrogen Inc., Seoul, Korea). The obtained sequences in this study were edited by MEGA software (version 6). After cutting the ambiguous regions and manual correction, the sequences were subjected to BLAST (http://www.ncbi.nlm.nih.gov/BLAST/) analysis to obtain the closely related sequences. 

The final edited sequences were subjected to BioEdit software (version 7.0.5) [ 17 ] for pairwise comparisons and determination of the levels of sequence difference counts in the nucleotides. A maximum-likelihood phylogenetic tree was constructed using the MEGA software (version 6) with the Tamura-Nei model and gamma-distributed substitution rate. The reliability for each group was evaluated by bootstrap analysis of 1000 replications, and a bootstrap support (bs) value of 70% was considered signiﬁcant. The *Penicillium* b-tubulin DNA sequences obtained in this study and their predicted amino acid sequences were deposited in GenBank under the accession numbers MN418400–MN418445. 

### Morphological analysis

To study the morphological characteristics, the fungi were three-point inoculated onto the media SDA, Czapek yeast autolysate agar (Quelab, Canada), malt extract agar (Sigma, Germany), and potato dextrose agar (Merck, Germany), in 8-cm plastic Petri dish. The plates were incubated at 25 °C and 37 °C (for malt extract agar) for 7-day cultures under dark conditions in the thermostatic incubator, and the colony morphology and degree of growth were studied. Moreover, microscope slides were prepared based on slide culture techniques and then micro morphological characters were examined using bright-field microscopy with a camera (Euromex, Holland). 

## Results

During a period of 12 months, 157 *Penicillium* isolates were screened for phenotypic colony characters, and 46 representative strains out of 11 morphological species were identified and selected for molecular analysis. In this study, the results of molecular identification showed that *P. crustosum* (18 isolates, 39.1%) and *P. chrysogenum* (15 isolates, 32.6%) were the most common isolated species, followed by *P. brevicompactum*, *P. rubens*, *P. citrinum*, *P. italicum* (each 2 isolates, 4.3%), *P. olsonii*, *P. expansum*, *P. griseofulvum*, *P. palitans*, and *P. polonicum* (each 1 isolate, 2.1%), in that order. 

**Figure 1:**
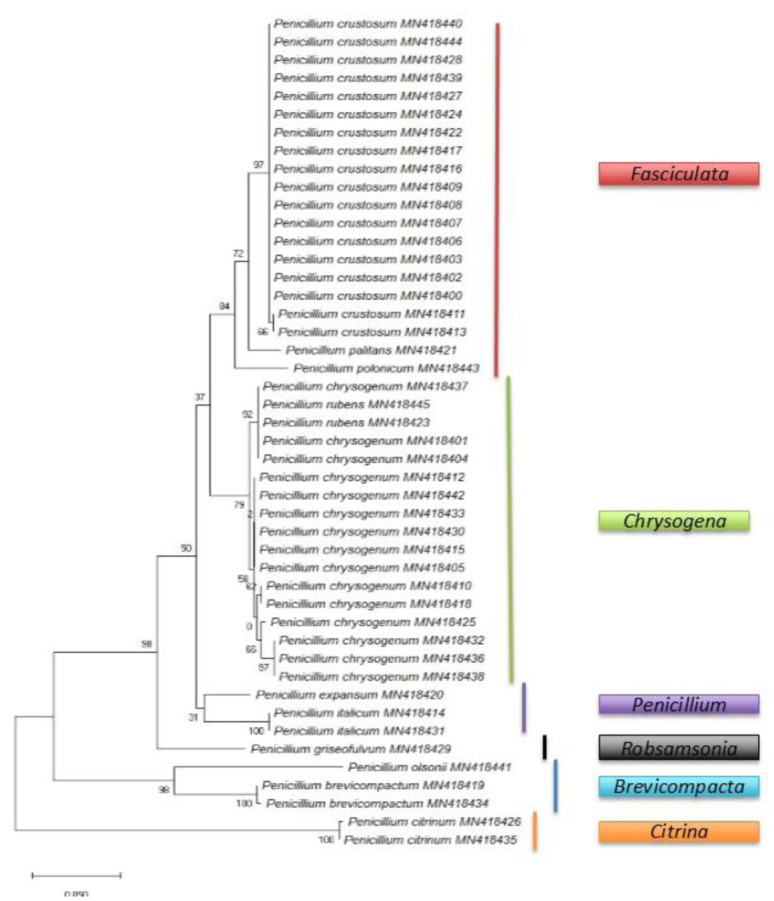
Phylogenetic tree of *Penicillium* species based on analysis of b-tubulin gene sequences. The evolutionary history was inferred using the maximum-likelihood method based on the Tamura-Nei model

Comparison of the DNA sequences showed intra-species differences of 0-1 (18 strains), 0-8 (15 strains), 0-1 (two strains), and 0-1nt (two strains) within strains of *P. crustosum*, *P. chrysogenum*, *P. brevicompactum*, and *P. citrinum*, respectively. However, *P. rubens* and *P. italicum* strains were invariant. Regarding the evolution and insights into the identification and taxonomy of species, the maximum likelihood phylogenetic tree of the b-tubulin DNA sequences of the 46 *Penicillium* species from the present study showed that our isolates belonged to six sections. These sections include *Fasciculata* (n=20), *Chrysogena* (n=17), *Brevicompacta* (n=3), *Penicillium* (n=3), Citrina (n=2), and *Robsamsonia* (n=1) (Figure 1). 

**Figure 2:**
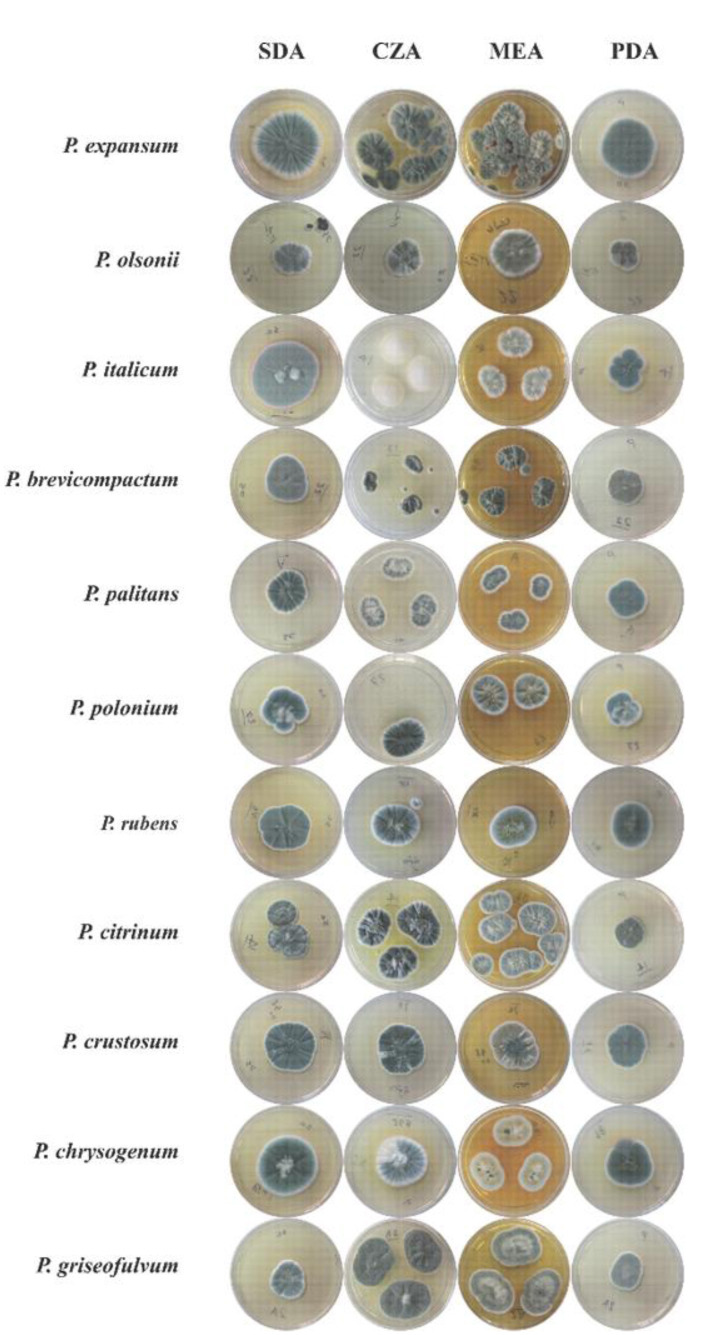
Colony morphology of *Penicillium* species on four different media (Sabouraud dextrose agar, Czapek Dox agar, malt extract agar, and potato dextrose agar)

**Figure 3:**
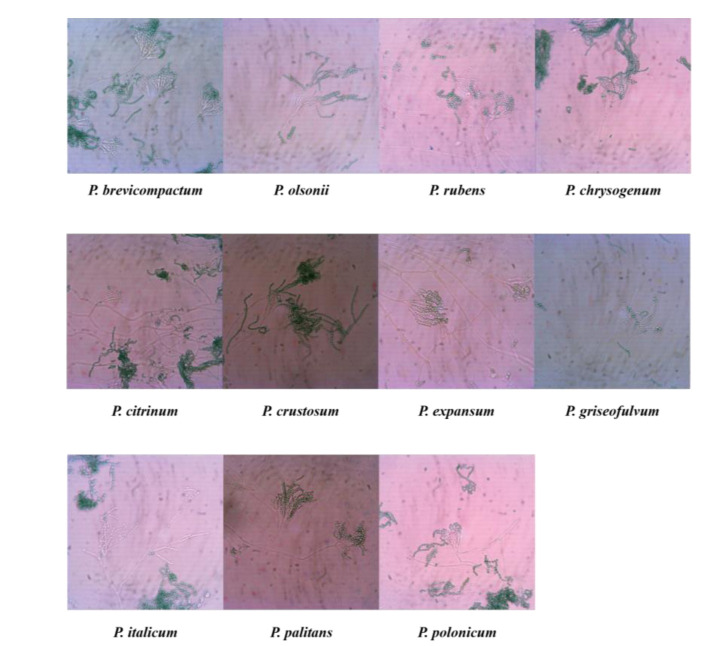
Micromorphology of different species of *Penicillium*

**Figure 4:**
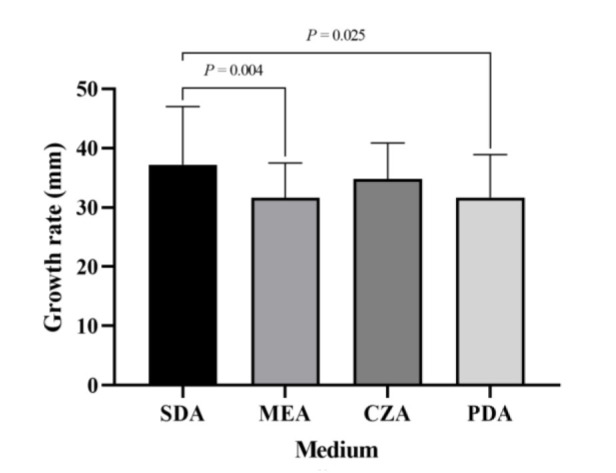
Comparison of colony growth of *Penicillium* species based on four different media

**Figure 5:**
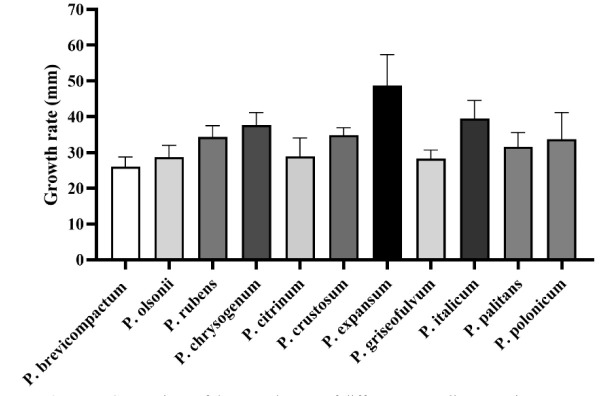
Comparison of the growth rates of different *Penicillium* species.

Given the nucleotide similarity between closely related species, in order to confirm the species, the strains were examined by macro-morphological characteristics based on phenotypic criteria. Figures 2 and 3 show the macro and microscopic features of *Penicillium* species. Except for P. chrysogenum and P. expansum with floccose colony texture, the rest of the species had velutinous texture. *P. chrysogenum* and *P. polonicum* conidiophores were defined as both biverticillate and terverticillate, while all other species had two-stage branched (terverticillate) conidiophores. The growth rate of *Penicillium* species was also compared for 7 days at 25 °C on four different culture media. Comparison of the data obtained from the Friedman analysis test confirmed that the maximum and minimum growth rate of colonies were observed on the SDA medium (Figure 4), and potato dextrose agar (PDA), respectively. There was a significant difference between the growth rate on SDA and that on PDA and malt extract agar (MEA) (P<0.018), while there was no significant difference between the growth rate on SDA and that on Czapek Dox agar (CZA) (P>0.05). The highest growth rates of *Penicillium spp*. were detected in *P. expansum* (P<0.0008), while the lowest growth rate was observed in *P. brevicompactum* (Figure 5).

## Discussion

The dynamic nature of aeroallergens in the atmosphere is obscurest and attributable to many different conditions, such as the time of day, geographic location, air pollution, weather conditions, human activities, and local vegetation resources [ 18 ]. Growth and sporulation of fungi may occur due to the long-term high humidity in a building [ 19 ]. Outdoor air is often the predominant source of indoor fungi, and fungal spores play an important role in plant pathology and various types of human health problems, including primary infections, allergic reactions, irritations, and toxic effects [ 20 - 22 ]. The information obtained from fungal air samples can be useful in medical evaluations, health risk assessment, and it can be useful in proactive indoor air monitoring [ 19 ]. Similarly, there is little published data evaluating outdoor fungal populations in different parts of Iran. 

*Penicillium* species are mainly mesophilic fungi (optimal temperature around 25 °C), and as fast-growing fungi produce a high number of airborne conidia that are easily dispersed by air [ 23 ]. Exposure to high levels of *Penicillium* spores is associated with an increased risk of respiratory symptoms in infants at risk for respiratory infections and asthma [ 24 , 25 ]. Due to the flexibility of morphological features of *Penicillium* species, precise identification is difficult [ 3 ]. Therefore, the reverse taxonomic strategy was used in which the species were identified using a molecular marker and then confirmed by the morphological approach [ 26 ]. 

In the present study, molecular identiﬁcation results showed that *P. crustosum* and *P. chrysogenum* were the most common isolated species, followed by *P. brevicompactum*, *P. rubens*, *P. citrinum*, *P. italicum*, *P. olsonii*, *P. expansum*, *P. griseofulvum*, *P. palitans*, and *P. polonicum*, in that order. According to Abastabar et al. [ 27 ], *Penicillium* species were isolated from soil, air, cereals, and decaying vegetable in Tehran and Mazandaran provinces in the north of Iran. They identified 10 species of this genus, including *P. chrysogenum*, *P. polonicum*, *P. canescens*, *P. griseofulvum*, *P. italicum*, *P. raistrickii*, *P. expansum*, *P. melanoconidium*, *P. palitans*, and *P. glabrum*.

In Iran, Sepahvand et al. investigated the 707 fungal colonies isolated from 20 different genera in the air and surfaces of Khoramabad Hospital, Khoramabad, Iran, and found *Penicillium* fungi as the most prominent fungal genus without precise identification of *Penicillium* species [ 28 ]. Moreover, Shams-Ghahfarokhi et al. studied airborne fungi using the settle plate method in the outdoor environment in Tehran and showed Penicillium species as the third most prevalent genus after *Aspergillus* and *Cladosporium* without accurate discrimination of different *Penicillium* species [ 29 ].

The molecular phylogeny derived from the sequence confirms species identification and highlights intra-specific diversity for some species i.e. *P. crustosum*, *P. chrysogenum*, *P. brevicompactum*, and *P. citrinum*. In addition, a previous study conducted by Sabokbar et al. [ 30 ] demonstrated that intra-species variation of *Penicillium* strains isolated from the air using RAPD-PCR without accurate identification of *Penicillium* species.

Phylogenetic analysis used in this study showed three species related to the Fasciculata section, including *P. crustosum*, *P. palitans*, and *P. polonicum*. In the present study, *P. crustosum* had yellow exudate on MEA media. This species with a wide range of secondary metabolites and several mycotoxins, including viridicatins, penitrems, roquefortines, and terrestric acids, are found in various sources, such as soil and air, which is important in regions as a contaminant of nuts, oilseeds, meat, and cheese [ 31 ]. 

Moreover, *P. palitans*, as a major producer of cyclopiazonic acid, can be found in meat and cheese products [ 4 ]. *P. polonicum* as an airborne fungus with a wide range of hosts, such as dried meats, cereals, onions, and peanuts, produces a variety of bioactive natural products with potential chemotherapeutic agents and antifungal, antibiotics, and anticancer characteristics [ 32 - 34 ].

In the *Chrysogena* section, *P. chrysogenum* and *P. rubens* were found. *P. chrysogenum* had a floccose colony texture, while *P. rubens* had a velutinous texture. Skin and invasive infections caused by *P. chrysogenum* have been previously reported [ 35 - 37 ] and *P. rubens*, the closely related species to *P. chrysogenum*, has been isolated from clinical specimens which should be carefully considered [ 38 ]. The floccose colonies and the observation of branched conidiophores, branched metulae, brush-like clusters, and large amounts of spherical conidia produced by species belonging to this section were useful for the identification of *P. chrysogenum* strain [ 2 , 39 ]. 

In the present study, the most identified species within the *Penicillium* section were *P. expansum*, and *P. italicum* which are phenotypically different. *P. expansum* with its blue-green and velutinous colony on MEA and PDA is the main and widespread causal agent of postharvest decay of pomaceous fruit worldwide that produces some mycotoxins, such as patulin, penicillic acid, and citrinin, while P. italicum produces rot on citrus fruits [ 4 , 40 - 42 ]. 

In the *Brevicompacta* section, *P. brevicompactum* and *P. olsonii* were identified. It should be mentioned that *P. brevicompactum* is widely distributed throughout the natural world and extensively used in clinical practices as it has great medicinal value. For instance, it is antiviral, antitumor, antibacterial, and antifungal and has the ability of fermentation production of Mycophenolic acid, while another species, *P. olsonii*, as an ochratoxin A producer, is able to grow at low temperatures [ 4 , 43 - 45 ].

It must be mentioned that *P. citrinum* belongs to the *Citrina* section and is known for its symmetrically biverticillate conidiophores, flask-shaped phialides, and small conidia [ 46 ]. This species affects the kidneys and the immune system by producing the mycotoxin citrinin on citrus fruits, wheat, and other cereals [ 47 , 48 ].

In this study, *P. griseofulvum* with gray-green colony belonged to section *Robsamsonia*, a globally distributed species that produces several fungal metabolites, such as patulin, roquefortin C, cyclophosphamide, and griseofulvin. It is noteworthy that lone cyclophosphamide could be a venture for the consumers in dry-cured meat products [ 2 , 49 ]. The present study is the first to provide information regarding the identification of a large number of *Penicillium* strains isolated from air samples in southern Iran.

## Conclusion

To the best of our knowledge, this is the first study to focus on *Penicillium spp*. in the south of Iran. As different genera have similar morphological characteristics, this study provided precise morphological and molecular characteristics that seem to be a very valuable tool for the identification and classiﬁcation of the genus *Penicillium*. The results of this descriptive study provided data that can improve our understanding of the outdoor air fungi and help to develop a database that includes information on the distribution of *Penicillium* in different places.

## Acknowledgement

The authors would like to thank the Research Deputy of Bushehr University of Medical Sciences, Bushehr, Iran, for their financial support (grant No. 20/71/751).

## Authors’ contribution

B. A., B. N., and G. K. designed and supervised all parts of the project and wrote the paper. Samples were collected by S. D. and F. F. Data collection and laboratory experiments were performed by I. M. and F. S. Data analysis was carried out by S. A. All authors approved the final version of the manuscript.

## Conflict of Interest

The authors declare no conflicts of interest. 

## Financial disclosure

The authors declare no financial disclosure.
